# Melanomas and Dysplastic Nevi Differ in Epidermal CD1c+ Dendritic Cell Count

**DOI:** 10.1155/2017/6803756

**Published:** 2017-02-26

**Authors:** Grzegorz Dyduch, Katarzyna Ewa Tyrak, Anna Glajcar, Joanna Szpor, Magdalena Ulatowska-Białas, Krzysztof Okoń

**Affiliations:** ^1^Chair of Pathomorphology, Faculty of Medicine, Jagiellonian University Medical College, Grzegórzecka 16, 31-351 Kraków, Poland; ^2^II Chair of Internal Medicine, Faculty of Medicine, Jagiellonian University Medical College, Skawińska 8, 31-066 Kraków, Poland

## Abstract

*Background.* Dendritic cells could be involved in immune surveillance of highly immunogenic tumors such as melanoma. Their role in the progression melanocytic nevi to melanoma is however a matter of controversy.* Methods.* The number of dendritic cells within epidermis, in peritumoral zone, and within the lesion was counted on slides immunohistochemically stained for CD1a, CD1c, DC-LAMP, and DC-SIGN in 21 of dysplastic nevi, 27 in situ melanomas, and 21 invasive melanomas.* Results.* We found a significant difference in the density of intraepidermal CD1c+ cells between the examined lesions; the mean CD1c cell count was 7.00/mm^2^ for invasive melanomas, 2.94 for in situ melanomas, and 13.35 for dysplastic nevi. The differences between dysplastic nevi and melanoma in situ as well as between dysplastic nevi and invasive melanoma were significant. There was no correlation in number of positively stained cells between epidermis and dermis. We did not observe any intraepidermal DC-LAMP+ cells neither in melanoma in situ nor in invasive melanoma as well as any intraepidermal DC-SIGN+ cells in dysplastic nevi.* Conclusion.* It was shown that the number of dendritic cells differs between dysplastic nevi, in situ melanomas, and invasive melanomas. This could eventually suggest their participation in the development of melanoma.

## 1. Introduction

Cutaneous malignant melanoma is an aggressive human neoplasm, characterized by constantly increasing prevalence rates and very high mortality. Additionally, despite advances in melanoma treatment, the available therapeutic agents are still not fully satisfactory for patients with advanced stages of the tumor. These facts are prompting scientists to search for new predictive markers and potential targets for melanoma therapy. Cutaneous malignant melanoma is considered to be one of the most immunogenic tumors [1] and it is suggested that circulating immune markers could correlate with the overall prognosis of the patient [2]. Dendritic cells (DCs), acting as professional antigen-presenting cells, play a key role in the tumor-associated immunological reactions. They are found in peripheral tissues and in immunological organs, displaying considerable heterogeneity in phenotype, location, and function [3–6]. On the basis of cell surface markers and intracellular molecules, different subtypes of skin DCs (Langerhans cells, dermal DC, plasmacytoid DC, and inflammatory dendritic epidermal cells) can be distinguished. The surface receptors of different DCs subtypes determine their ability to recognize, capture, and present tumor-associated antigens to naïve T cells in the context of major histocompatibility molecules (MHCs), providing a bridge between innate and adaptive immune responses [3, 7]. However, not only do immature DCs fail to stimulate naïve T cells to develop into effective CD4+ or CD8+ lymphocytes, but they also activate CD4+ CD25+ regulatory T cells, which enhance the unresponsiveness of effector T cells in the tumor microenvironment [8].

The role of DCs in development and propagation of various human cancers including breast, colon, esophageal, lung, and oral carcinoma [9–14] has been previously described. However, the role of dendritic cells and their cutaneous subtypes in the evolution of primary malignant melanoma remains to be confirmed as there are very few publications addressing this issue. In the previous studies the differences in distribution and phenotype of dendritic cells in intra- and peritumoral area have been observed. Most of the DC subsets found in melanoma cell nests were immature, suggesting defects in maturation process of melanoma-associated dendritic cells [15–18]. Moreover, the degree of infiltration by certain DCs has been considered to inversely correlate with melanoma thickness [16].

Beside their possible prognostic value, evaluation of dendritic cells might prove useful in designing new cell-based immunotherapy for patients' with advanced stages of cutaneous melanoma [19]. The advances in identification of tumor antigens facilitated the development of targeted treatment and immunomodulation therapy, including the use of patient's dendritic cells. Such treatment is based on increasing the capacity of the immune system to induce tumor regression, where dendritic cells play a crucial role, stimulating T cells to effective response against tumor-associated antigens [19, 20]. Over the last few years ex vivo-generated DCs [21] and in vivo-DC-targeting [22] have been widely investigated as a potential therapeutic vaccine in various types of cancers [23]. In humans, Food and Drug Administration has already approved the targeted DC-based immune therapy (sipuleucel-T vaccine) for prostate cancer [6].

In the present study, we aimed to investigate the density of dendritic cells expressing CD1a, CD1c, DC-SIGN, and DC-LAMP. CD1a and CD1c are nonclassical MHC class I antigens [24]; the expression of CD1a is largely confined to DCs of the human epidermis, whereas the presence of CD1c has been observed in both dermal and epidermal cells [23]. DC-SIGN is the C-type lectin receptor, regulating adhesion processes, such as DC trafficking, transient T-cell binding, and antigen capture [21, 25]. The expression of DC-LAMP, a lysosome-associated membrane glycoprotein, defines mature dendritic cells. The samples were obtained from 69 patients with one of the following diagnosis: dysplastic nevus, melanoma in situ, and invasive melanoma (level III or IV according to Clark scale). DC density results were evaluated with regard to the type of the melanocytic lesion, dendritic cells' surface markers, and maturation status as well as their association with clinical characteristic of a patient. The obtained results might prove useful in explaining the prognostic and predictive significance of dendritic cells infiltrating different melanocytic lesions.

## 2. Materials and Methods

### 2.1. Tissue Specimens

We analysed 69 cutaneous samples obtained between 2005 and 2014 from patients who were diagnosed with dysplastic nevus, melanoma in situ, or invasive melanoma. The paraffin-embedded samples were obtained from the archives of the Department of Pathomorphology. Data concerning localization of the lesions as well as patients' sex and age were collected from the referrals for the histopathological examination stored in the database of the Department of Pathomorphology.

### 2.2. Immunohistochemistry

Immunohistochemistry was performed using the following monoclonal antibodies: CD1a, CD1c, DC-LAMP, and DC-SIGN. Primary antibodies and dilution as well as the retrieval procedure used in our study are summarized in Table 1. Cutaneous tissue samples were stained manually and processed according to the protocol used on a routine basis in the laboratory of the Department of Pathomorphology. The selected paraffin-embedded tissue blocks were cut into 4 *μ*m thick sections, mounted on SuperFrost glass slides (Thermo Scientific, USA), and dried in an incubator for 12 hours in 34°C. The obtained slides were deparaffinized, dehydrated, and then incubated in 3% H_2_O_2_ solution for 10 minutes to block endogenous peroxidase activity. Antigen retrieval was performed by immersing the slides in citrate buffer (pH 6.0; 0.01 M) or EDTA (pH 8.0; 0.01 M) and subjecting them to 97°C in a water bath for 30 minutes.

Polyclonal secondary antibodies conjugated to horseradish peroxidase (HRP) enzyme (Ultra Vision LP Value Detection System HRP Polymer, Lab Vision, Thermo Scientific, USA) were applied to visualize the obtained antigen-antibody complexes, using DAB (3,3′-diaminobenzidine) as chromogen. Cell nuclei were stained with hematoxylin to enhance contrast in tissue sections.

### 2.3. Evaluation of Immunostaining

Quantitative assessment of each DC subset was performed in light microscopy on the basis of the numbers of positively stained cells (membrane and cytoplasmic brown staining). The slides were firstly examined at low magnification (10x lens) to select “hot spots” of positively stained cells. Secondly, the number of positively stained cells was counted at high magnification (40x lens) and expressed per 1 mm^2^. The number of dendritic cells was evaluated in epidermis and in dermis separately, for each monoclonal antibody independently in every cutaneous sample. In epidermis cells were counted above the tumor tissue, in dermis in 0.5 mm band of peritumoral tissue, or beneath epidermis (in situ lesions). In dysplastic nevi and invasive melanoma cases positively stained cells were also counted in within and between nests of melanocytes (intratumoral location). The obtained results were entered into Microsoft Office Excel Spreadsheet (Microsoft Corporation, USA).

### 2.4. Statistical Analysis

Statistical data analysis was performed using Statistica 10 software (StatSoft, USA). Relationships between the presence of each of the DC subsets and patient's sex were evaluated by using Mann–Whitney* U* test. Spearman rank correlation was performed to assess correlations between the numbers of respective DC subsets. ANOVA Kruskal-Wallis test was used to evaluate the relationships between the content of respective DC subsets and the type of melanocytic lesion. *p* values ≤ 0.05 were considered significant.

## 3. Results

The study group is comprised of 37 women and 32 men. The mean age of patients at the time of diagnosis was 50.9 years (ranging from 6 to 81 years).

Among 69 cases included in our study 21 (30.43%) were classified as dysplastic nevi (compound, with moderate-to-severe atypia), 27 (39.13%) as melanoma in situ, and 21 (30.43%) as invasive melanoma. The mean tumor thickness in invasive melanoma group was 1.78 mm (ranging from 0.5 mm to 5.6 mm).

There was no significant association between the ratio of positively stained DCs (both in epidermis and in dermis) and the sex as well as the age of a patient in any of the examined DC subsets. There was also no correlation in number of positively stained cells compared between epidermis and dermis in each DC subset independently (*p* > 0.05 for all the markers).

**Figure 1:**
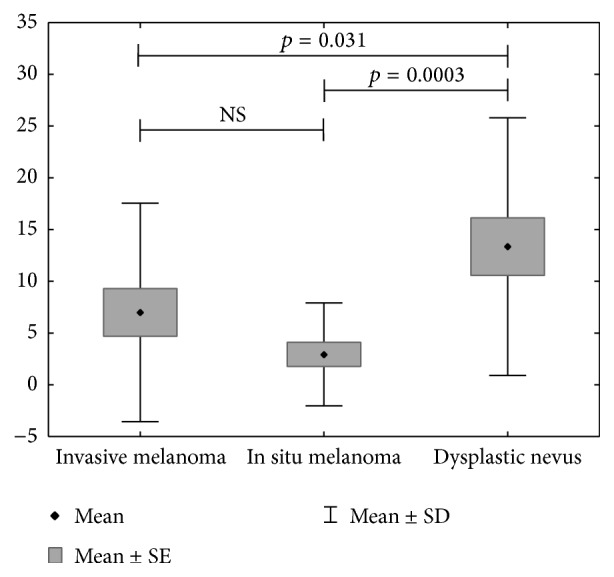
Density of CD1c+ epidermal dendritic cells in nevi, in situ, and invasive melanomas.

**Figure 2:**
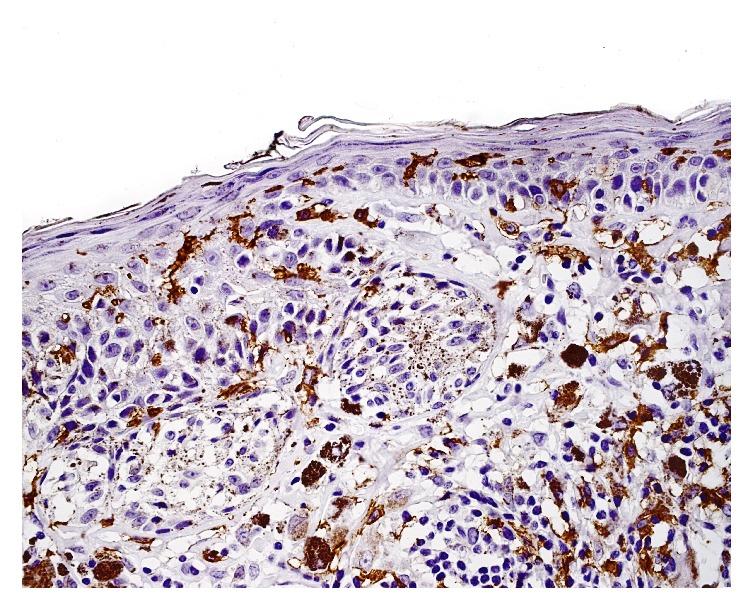
Dysplastic nevus. CD1c+ dendritic cells seen in epidermis and dermis.

**Figure 3:**
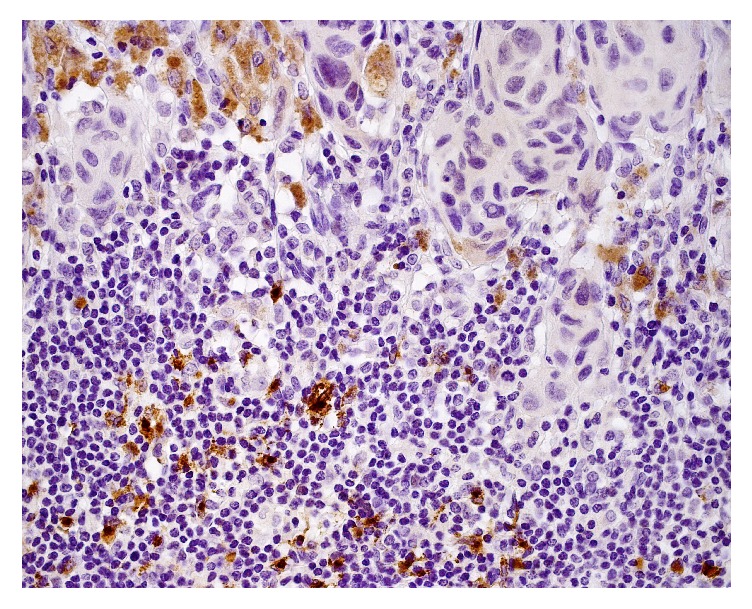
Invasive melanoma. DC-LAMP+ cells seen in dermis, beneath the tumor mass.

Mean value of CD1c^+^ cells was significantly higher in dysplastic nevi in comparison with both melanoma in situ and invasive melanoma. The difference in intraepidermal CD1c^+^ cells number (*p* = 0.0003) between examined tissues was statistically significant. The differences were observed between dysplastic nevi and melanoma in situ (*p* = 0.0003) as well as between dysplastic nevi and invasive melanoma (*p* = 0.031) (Figure 1). Mean value of CD1c^+^ cells was significantly higher in dysplastic nevi in comparison with both melanoma in situ and invasive melanoma. No significant differences were found in the content of DCs between the analysed types of melanocytic lesions in case of CD1a^+^ cells (intraepidermal and dermal), CD1c^+^ in dermis, DC-LAMP^+^ (intraepidermal and dermal), and DC-SIGN^+^ (intraepidermal and dermal). CD1a+ and CD1c+ cells were detected both in epidermis and in dermis in all types of investigated melanocytic lesions (Figure 2). We did not observe any intraepidermal DC-LAMP+ cells neither in melanoma in situ nor in invasive melanoma (Figure 3) as well as any intraepidermal DC-SIGN+ cells in dysplastic nevi (Table 2). There were no significant differences between dysplastic nevi and invasive melanomas regarding number of DCs of any subset in intratumoral location. In that location mean values of dendritic cell number in dysplastic nevi versus invasive melanomas were, respectively, 14.72 versus 16.82 for CD1a, 32.00 versus 35.20 for CD1c, 1.0 versus 0.82 for DC-LAMP+, and 3.22 versus 4.00 for DC-SIGN+.

## 4. Discussion

DCs have attracted attention in recent years due to their established prognostic and predictive value in various diseases [26, 27] as well as potential usability in immunotherapy strategies [19, 28–31]. Several authors have investigated DC infiltration in cutaneous malignant melanoma samples showing decreased number in advanced stages of the tumor [16, 17, 32]. The others have noted a correlation of the plasmacytoid DCs subset with tumor thickness and poor prognosis [15, 33] and a positive correlation of density of DC-LAMP+ mature DCs with survival in univariate analysis [15]. Nonetheless, there are very few up-to-date studies investigating the significance of DCs in neoplastic melanocytic skin lesions, quantifying various subsets and relative contribution of epidermal and dermal DCs to host immunosurveillance. Literature is also lacking in data concerning the infiltration of dysplastic nevi by dendritic cells.

In our cohort of melanocytic lesion samples, we found a statistically significant increase in the number of CD1c-positive cells in the epidermis of melanoma in situ and invasive tumors when compared with dysplastic nevi. Other authors have also obtained similar results, showing the depletion of intraepidermal DCs in melanoma [17, 32]. However, it should be mentioned that these other studies looked at the DC number in general, as at that time the available immunochemistry was insufficient to precisely quantify distinct DC subsets. The reason why these differences are restricted to epidermis might be partly explained by distinct origin and features of DCs in respective layers of the skin. Langerhans cells (LCs), representing the DC population found in epidermis, are considered to be of nonlymphoid tissue origin, while dermal DCs derive from bone marrow and blood-borne precursors [34]. The lack of blood vessels in epidermis together with the presence of basement membrane may also be contributory. Moreover, it is suggested that some melanoma-derived factors might adversely affect epidermal Langerhans cells' migration, differentiation, and function [32]. For instance, TGF-beta and IL-10, found in melanoma cell nests, have been described to reduce the development of DCs [34, 35]. In addition, necrosis, regression, or scarring of the tumor in more advanced stages may contribute to the depletion of DCs in epidermis overlying this type of malignant skin lesions [32]. Our finding indicates that epidermal area of melanomas might not be adequately equipped with CD1c-positive DC subset and thereby might be unable to elicit an appropriate antitumor immune response. However, whether the CD1c-positive cells depletion occurs during the initiation, promotion, or progression of the tumor calls needs further elucidation. There is also little available data in the literature with regard to CD1c antibody and the exact pathophysiological significance of this observation remains to be established.

CD1a-positive DCs have been widely investigated in various human cancers with results varying between papers. We did not observe any difference in CD1a-positive cells density between epidermis and dermis in any of the investigated types of melanocytic lesions. However, Ladányi et al. have shown reduced number of intraepidermal CD1a-positive Langerhans cells overlying melanomas, suggesting that it could be explained by the fact that even nonulcerated epidermis above melanoma is often atrophic or hyperkeratotic. It has also been reported that infiltration of CD1a-positive DC within tumor cell nests and peritumoral area correlates with decreased tumor thickness and the radial growth phase of melanomas, respectively [16]. Vermi et al. observed the significant increase in CD1a-positive cells accumulation in primary cutaneous melanoma compared with normal skin and melanocytic nevi; in melanoma samples immature CD1a-positive DCs were mainly found in the dermis surrounding the tumor, in close vicinity of lymphoid cell aggregates [18]. Recently, presence of CD1a-positive DCs around the tumor cell nests was also observed to correlate with the absence of hematogenous spread in malignant melanoma of the skin [36]. In other human malignancies the decreased number of CD1a-positive DCs in tumor area is often associated with the progression of the disease and unfavourable prognosis [11, 14].

DC-SIGN-positive DCs have recently emerged as very potent cells shaping immune responses by mediating immune recognition and cell adhesion [37]. Despite the fact that our study did not reveal any statistically significant differences in the density and distribution of this subset, noteworthy is very low epidermal count of DC-SIGN-positive DCs, as we have observed a few of them in only two samples among all the investigated cases. We think that this subset of DCs requires further investigation on a larger sample of cases, especially as data concerning this marker in melanocytic lesions is scarce and ambiguous. Although DC-SIGN antibody is generally classified as nonspecific marker of DC maturation [38], in one study on malignant melanoma the expression of DC-SIGN and DC-LAMP was found to be mutually exclusive, indicating that DC-SIGN is expressed only by immature DCs. At the same time, DC-SIGN-positive dermal DCs were noted to encompass distinct subset compared to immature CD1a-positive DCs. It should also be born in mind that DC-SIGN antibody may stain positively other immune cells, such as certain subsets of macrophages [18].

We have also noted the lack of mature DC-LAMP-positive dendritic cells in epidermis of melanoma in situ and invasive tumors and very low number of them in dermal compartment. This is in agreement with findings from other studies concerning various cancers, which highlight a suppressive tumor influence on DCs function, as the infiltrating cells in advanced stages of the disease are mostly immature and thus unable to present effectively tumor-derived antigens and mediate antitumor response [39–41]. The content of DC-LAMP-positive DCs in primary cutaneous melanoma was also found to be rather scarce and mostly confined to the peritumoral lymphocyte infiltrate [15]. Mature DCs were observed to form clusters with T cells, which could be an evidence of a still ongoing immune response in tumor microenvironment. It has also been shown that the degree of DC-LAMP+ cells infiltration inversely correlates with melanoma thickness and is associated with patients' survival [16].

The present study investigated only some problems concerning the density of DCs in dysplastic and malignant melanocytic lesions. It is still unclear whether the differences in DC content and distribution patterns are a cause or a result of neoplastic processes. Another interesting question may arise with regard to the DCs subsets, whose density and location do not differ significantly between distinct melanocytic lesions, regardless of whether they are of malignant nature or not. Do DCs fail to serve as part of host immune system control due to some impairment of functioning or should it be rather attributable to the low immunogenicity of melanoma cells? Undoubtedly, a thorough analysis of dendritic cell family, including a variety of markers and double staining procedures, is mandatory to draw valid conclusions on the development of immunity in the carcinogenesis of malignant melanoma. Moreover, analysis of a large number of melanoma samples along with the prospective study of clinical data could offer better understanding of predictive and prognostic significance of tumor-infiltrating DCs. Although our study requires further validation, we believe that it sheds new light on the role of DCs in dysplastic and neoplastic cutaneous lesions and thus brings added value to the optimal patient care and development of novel immunotherapy strategies.

## Figures and Tables

**Table 1:** Antibodies used in the study.

Primary antibody	Clone	Dilution	Antigen retrieval	Incubation time	Producer
CD1a	MTB1	1 : 10	Citrate	Overnight	Novocastra (Leica Biosystems, Germany)
CD1c	5B8	1 : 200	EDTA	30 min	Abcam, UK
DC-LAMP	Rabbit polyclonal	1 : 50	EDTA	30 min	Novus, USA
DC-SIGN	5D7	1 : 50	EDTA	30 min	Abcam, UK

**Table 2:** Number of dendritic cells in epidermal and dermal compartment of dysplastic nevi and in situ and invasive melanomas.

	Epidermal				Dermal			
	Mean	Range	SD	*p*	Mean	Range	SD	*p*
CD1a								
Invasive melanoma	38.24	0–80	21.63	0.2808 (NS)	22.55	0–53	14.79	0.2434 (NS)
In situ melanoma	35.84	15–70	13.25		16.32	3–39	10.85	
Dysplastic nevus	43.29	18–74	14.92		15.29	1–43	11.27	
*All cases*	*39.23*	*0–80*	*17.10*		*18.03*	*0–53*	*12.65*	

CD1c								
Invasive melanoma	7.00	0–36	10.55	0.0003	51.81	6–96	30.79	0.0559 (NS)
In situ melanoma	2.94	0–17	4.96		33.56	0–151	37.21	
Dysplastic nevus	13.35	2–45	12.44		40.00	8–172	35.08	
*All cases*	*7.92*	*0–45*	*10.70*		*42.24*	*0–172*	*34.56*	

DC-LAMP								
Invasive melanoma	0.00	0-0	0.00	0.3588 (NS)	1.26	0–10	2.62	0.2456 (NS)
In situ melanoma	0.00	0-0	0.00		0.77	0–14	3.02	
Dysplastic nevus	0.05	0-1	0.22		0.85	0–7	2.01	
*All cases*	*0.02*	*0-1*	*0.13*		*0.95*	*0–14*	*2.57*	

DC-SIGN								
Invasive melanoma	0.10	0–2	0.45	0.5867 (NS)	11.89	0–39	13.54	0.2495 (NS)
In situ melanoma	0.26	0–5	1.15		4.53	0–26	8.19	
Dysplastic nevus	0.00	0-0	0.00		4.24	0–32	7.31	
*All cases*	*0.12*	*0*–*5*	*0.69*		*6.71*	*0–39*	*10.34*	
